# Microstructural and Physicochemical Analysis of Collagens from the Skin of Lizardfish (*Saurida tumbil* Bloch, 1795) Extracted with Different Organic Acids

**DOI:** 10.3390/molecules27082452

**Published:** 2022-04-11

**Authors:** Abdul Aziz Jaziri, Rossita Shapawi, Ruzaidi Azli Mohd Mokhtar, Wan Norhana Md. Noordin, Nurul Huda

**Affiliations:** 1 Faculty of Food Science and Nutrition, Universiti Malaysia Sabah, Kota Kinabalu 88400, Malaysia; azizjaziri@ub.ac.id; 2 Faculty of Fisheries and Marine Science, Universitas Brawijaya, Malang 65145, Indonesia; 3 Borneo Marine Research Institute, Universiti Malaysia Sabah, Kota Kinabalu 88400, Malaysia; rossita@ums.edu.my; 4 Biotechnology Research Institute, Universiti Malaysia Sabah, Kota Kinabalu 88400, Malaysia; ruzaidi@ums.edu.my; 5 Fisheries Research Institute, Batu Maung, Penang 11960, Malaysia; wannorhana@yahoo.com

**Keywords:** lizardfish skin, acid-extracted collagen, structural property, physicochemical characteristics

## Abstract

Marine fish collagen has attracted considerable attention due to its characteristics, including its biodegradability, biocompatibility, and weak antigenicity, and is considered a safer material compared to collagen from terrestrial animals. The aim of this study was to extract and characterize collagen from the skin of lizardfish (*Saurida tumbil* Bloch, 1795) with three different acids. The yields of acetic acid-extracted collagen (AESkC), lactic acid-extracted collagen (LESkC), and citric acid-extracted collagen (CESkC) were 11.73 ± 1.14%, 11.63 ± 1.10%, and 11.39 ± 1.05% (based on wet weight), respectively. All extracted collagens were categorized as type I collagen with mainly alpha chains (α_1_ and α_2_) detected and γ and β chains to some extent. Fourier transform infrared (FTIR) spectra showed an intact triple-helical structure in the AESkC, LESkC, and CESkC. UV-vis spectra and X-ray diffraction further demonstrated the similarity of the extracted collagens to previously reported fish skin collagens. AESkC (*T_max_* = 40.24 °C) had higher thermostability compared to LESkC (*T_max_* = 38.72 °C) and CESkC (*T_max_* = 36.74 °C). All samples were highly soluble in acidic pH and low concentrations of NaCl (0–20 g/L). Under field emission scanning electron microscopy (FESEM) observation, we noted the loose, fibrous, and porous structures of the collagens. The results suggest that the lizardfish skin collagens could be a potential alternative source of collagen, especially the AESkC due to its greater thermostability characteristic.

## 1. Introduction

Biopolymers such as collagen, elastin, hyaluronic acid, and heparin derived from natural sources have a great prospective. Because of their special traits such as biocompatibility, biodegradability, and the ability to work with various cell adhesives and differentiation-promoting motifs. In addition, they have been approved by the FDA and are already being used in clinics [1,2,3]. Collagen is a fibrous protein and a major component in the connective tissues of animals, representing about one third of the total protein composition [4]. It is structurally characterized as a unique right-handed triple helix, comprised of three left-handed helical polypeptide chains, each with a (Gly-X-Y) repeating sequence where X and Y are often proline and hydroxyproline [5]. At present, approximately 29 types of collagens have been investigated and each type has a unique protein structure, amino acid sequence, and biophysical characteristic [6]. Type I collagen is the most abundant collagen in the skin, bone, and tendon [7,8]. This type of collagen is widely used in the food and beverages, cosmetic, nutraceutical, pharmaceutical, and biomedical industries because of its excellent properties, including biocompatibility, biodegradability, and weak antigenicity [9,10]. Traditionally, the main sources of commercial collagen were derived from terrestrial animals, such as bovine and porcine. However, the use of bovine collagen is not well accepted because of its association with bovine spongiform encephalopathy (BSE), transmissible spongiform encephalopathy (TSE), and foot-and-mouth disease (FMD) [11]. In addition, the use of bovine collagen is also an issue for Hindus and Sikhs, while porcine collagen is prohibited to Muslims and Jews [12]. Therefore, alternative sources of collagen are needed.

For the last few years, fish collagen has drawn considerable attention amongst researchers and processors. Fish collagen has been demonstrated to be comparable to terrestrial animal collagen in reference [13]. Besides, it is proven as safe and acceptable to most religious beliefs. Hence, numerous studies have been initiated on the extraction of collagen from fish species, such as black ruff (*Centrolophus niger*), marine eel (*Evenchelys macrura*), miiuy croaker (*Miichthys miiuy*), silver catfish (*Pangasius* sp.), bigeye tuna (*Thunnus obesus*), carp (*Cyprinus carpio*), threadfin bream (*Nemipterus japonicus*), puffer fish (*Lagocephalus inermis*), giant grouper (*Epinephelus lanceolatus*), tilapia (*Oreochromis niloticus*), channel catfish (*Ictalurus punctatus*), and rohu (*Labeo rohita*) [14,15,16,17,18,19,20,21,22,23,24]. Additionally, their physicochemical properties, including thermostability, solubility, electrophoretic pattern, and structural and morphological analysis have been investigated. Most of the collagen extracted was obtained from by-products (skin, bone, scale, and fin) of the fish-processing industries. The extraction method is an important process to effectively remove collagen from fish by-products. The most common method used in collagen extraction is the acid-aiding technique [25]. Usually, organic acids such as acetic, lactic, and citric acid were used to extract fish collagen. These acids are effective in solubilizing the non-collagen chains and other internal chains compared to inorganic acids, such as hydrochloric acid. Besides, organic acids produced higher yields in a shorter extraction time, with lower cost, and were relatively safer [26]. Previous studies have described successful extractions of collagens using organic acids from various fish species [26]. 

Lizardfish (*Saurida tumbil* Bloch, 1795), also known as conor among the locals in Malaysia, is a commercially important marine fish for surimi processing. It belongs to the Synodantidae family, with adult fish lengths ranging from 19 cm to 35 cm. Its body is commonly brown above and silver below, and black with faint crossbands [27]. The average production of this fish in Malaysia was around 48,153 metric tons from 2015 to 2019 [28]. During fish processing, a large quantity of fish by-products is generated (60–75% of whole raw-fish weight) and is usually utilized for low-value-added products or sometimes discarded as waste, resulting in environmental pollution. Hence, the under-utilization of by-products has not only led to the loss of potential revenues but has also resulted in extra costs when disposing of these products [29,30]. The proper utilization of by-products is necessary to produce high-value products to bring financial gain and reduce environmental pollution.

Early works on the extraction of collagen from lizardfish have been reported by Moniruzzaman et al. [31], which focus on fish scale, and Taheri et al. [32] focus on gelatin from the lizardfish’s skin and bone. However, research on the extraction of collagen from lizardfish skin is much less explored to date. Our earlier study demonstrated that the skin of lizardfish had a higher protein content than other by-products (including scale, bone, and fins) [29]. Therefore, this study was carried out to extract collagen from the skin of lizardfish using three different organic acids and to evaluate its microstructural and physicochemical characteristics.

## 2. Results

### 2.1. Yield and Hydoxyproline Content

The yields of collagens from the skin of lizardfish (*S. tumbil*) extracted using acetic, lactic, and citric acid were 11.73 ± 1.14%, 11.63 ± 1.10%, and 11.39 ± 1.05% (based on the wet weight of the skin), respectively (Table 1). The yield from AESkC was slightly higher than the LESkC and CESkC although not significantly different (*p* > 0.05). 

Hydroxyproline (Hyp) is the main component of the imino acid that stabilizes the triple-helical structure of collagen. Hyp content could be used to measure collagen quantitatively since it is present almost exclusively in collagen. The Hyp (mg/g) from lizardfish skin collagens was initially determined and the total collagen (mg/g) was subsequently multiplied with 7.7 as a conversion factor, as described in the method of Kittiphattanabawon et al. [15]. The results demonstrated that the highest Hyp content was significantly detected in the AESkC sample (106.07 ± 0.13 mg/g), followed by the LESkC sample (82.42 ± 0.21 mg/g), and the CESkC sample (80.34 ± 0.46 mg/g). The AESkC sample showed the highest amount of collagen (*p* < 0.05) compared to other extracted collagens (Table 1). 

### 2.2. Color Analysis

Table 1 presents the color attributes (L*, a*, b*, and whitening index) of lizardfish skin collagens. The L* (brightness) was significantly highest in CESkC (*p* < 0.05) in comparison to the AESkC and LESkC. Furthermore, the highest whiteness index (WI) (*p* < 0.05) of lizardfish skin collagens was exhibited in the CESkC sample. Meanwhile, the a* and b* values of the extracted collagens were higher in the LESkC than in the AESkC and CESkC. 

### 2.3. UV Absorption Spectrum

**Figure 1:**
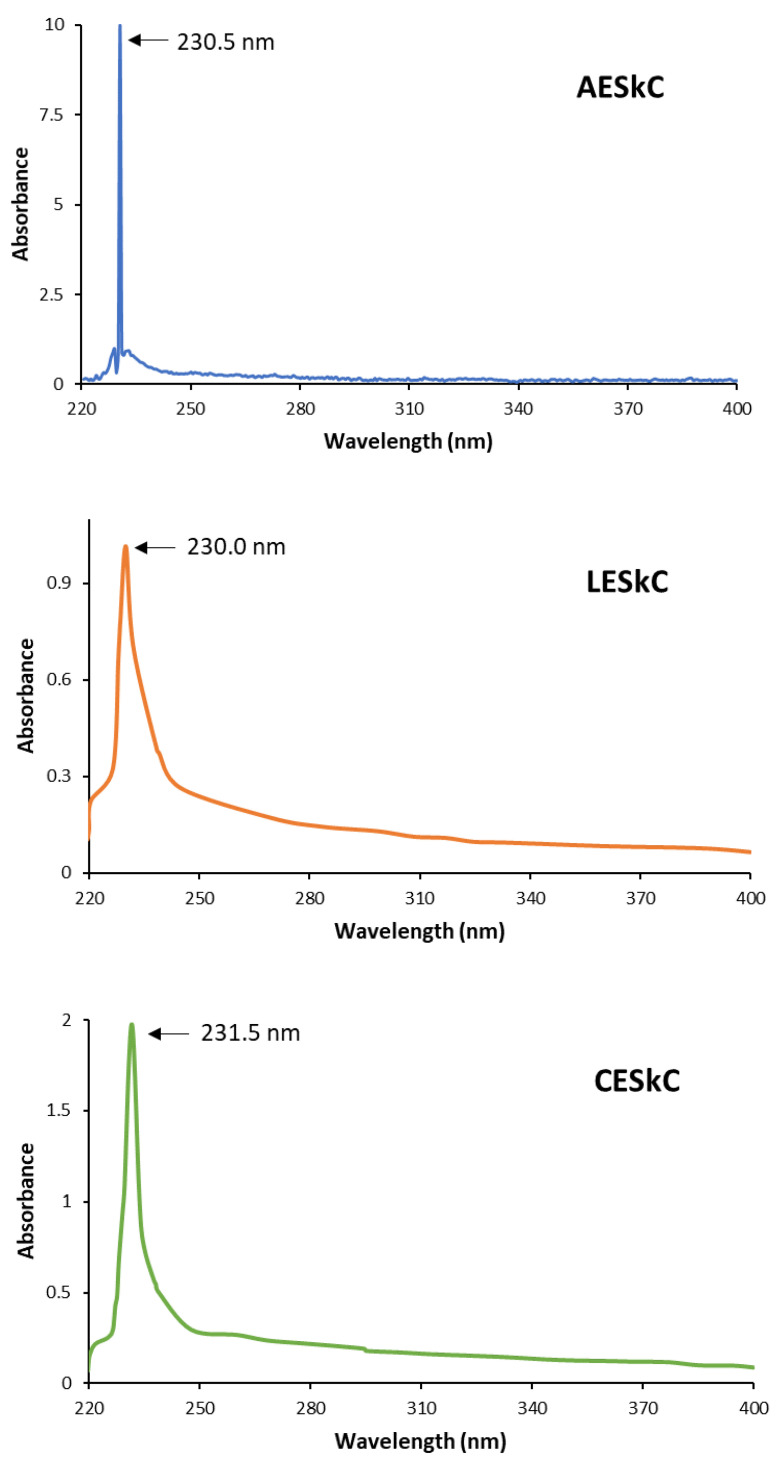
Ultraviolet absorption spectrum of collagens from the skin of lizardfish. AESkC: acetic acid-extracted collagen; LESkC: lactic acid-extracted collagen; CESkC: citric acid-extracted collagen.

In general, collagen could be characterized using the UV-vis spectra in the absorption wavelength of 220 nm to 240 nm, which represents the presence of C=O, -COOH, and CO-NH_2_ groups in the collagen triple helix [14]. As shown in Figure 1, AESkC, LESkC, and CESkC exhibited prominent peaks at 230.5 nm, 230.0 nm, and 231.5 nm, respectively. 

### 2.4. Attenuated Total Reflectance–Fourier Transform Infrared Spectroscopy (ATR–FTIR)

**Figure 2:**
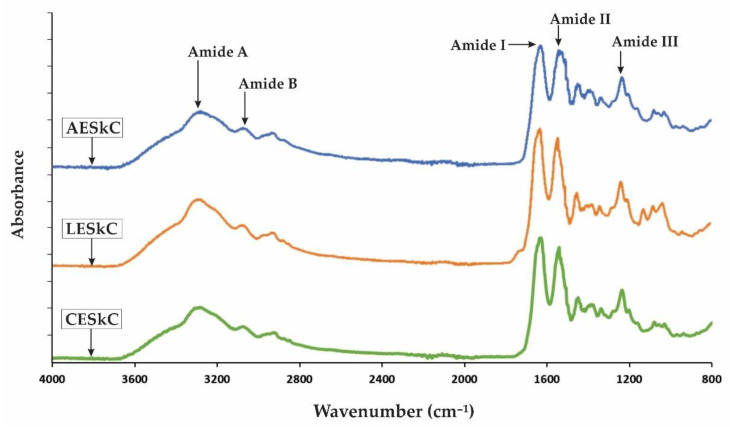
Fourier transform infrared spectroscopy peak locations and the assignment for collagens from the skin of lizardfish. AESkC: acetic acid-extracted collagen; LESkC: lactic acid-extracted collagen; CESkC: citric acid-extracted collagen.

The Fourier transform infrared spectroscopy was conducted at a wavelength that ranged from 4000 cm^−1^ to 400 cm^−1^, and the spectra is shown in Figure 2. Similar FTIR spectra were projected by AESkC, LESkC, and CESkC. The main absorption peaks, such as amide A, amide B, amide I, amide II, and amide III, could be observed in the amide region and were clearly assigned in Table 2. 

### 2.5. Sodium Dodecyl Sulfate-Polyacrylamide Gel Electrophoresis (SDS-PAGE)

**Figure 3:**
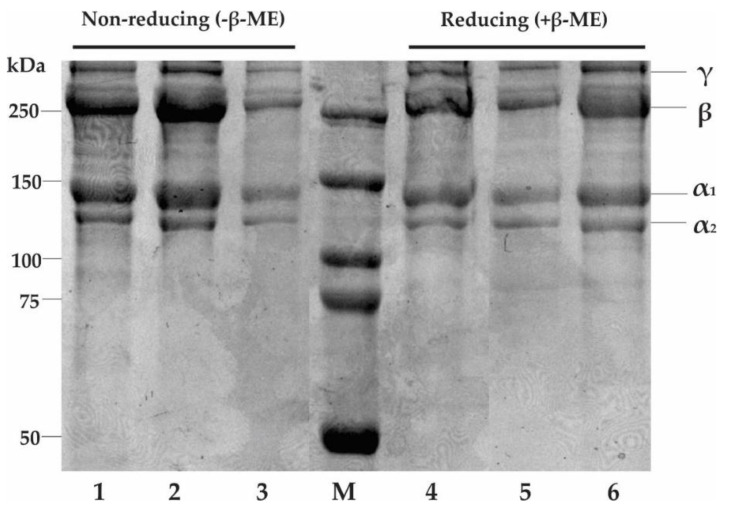
SDS-PAGE electrophoretogram of collagens from the skin of lizardfish shows the occurrence of band pattern of α, β, and γ isomers. Lane 1 and 4: acetic acid-extracted collagen (AESkC); lane 2 and 5: lactic acid-extracted collagen (LESkC); lane 3 and 6: citric acid-extracted collagen (CESkC); M: protein marker.

An SDS-PAGE analysis of collagen is widely used to indicate the type of collagen, subunit composition, band intensity, and electrophoretic mobility. Figure 3 illustrates the protein patterns of AESkC, LESkC, and CESkC treated with and without β-mercaptoethanol (β-ME). Similar protein patterns were observed with two main different alpha chains (α1 and α2) with molecular weights (MW) of around 139.61 and 123.95 kDa, respectively. In addition, the high MW in the γ and β chains were also detected in all samples. In terms of band intensity, the ratio of the α1 and α2 chains in all collagen samples was approximately 2:1. No difference in band positions between non-reducing and reducing treatment was observed in the extracted collagens.

### 2.6. X-ray Diffraction (XRD) Analysis

**Figure 4:**
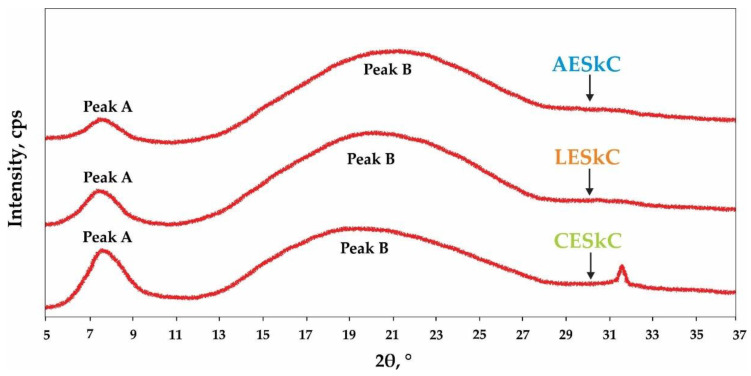
X-ray diffraction diagram of lizardfish skin collagens extracted with different acids. AESkC: acetic acid-extracted collagen; LESkC: lactic acid-extracted collagen; CESkC: citric acid-extracted collagen.

Collagen diffraction patterns are presented in Figure 4. There were two diffraction peaks at diffraction angles (2θ) exhibited in all lizardfish skin collagens. The diffraction peaks were mainly located at 7.40–7.63° and 19.17–20.86° with a *d*-spacing of 11.87–1192 Å and 4.25–4.63 Å, respectively.

### 2.7. Thermal Stability Study

**Figure 5:**
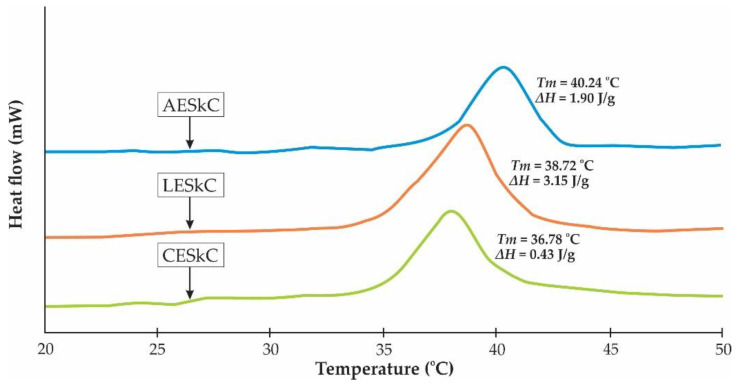
DSC thermogram of lizardfish skin collagens extracted with different acids. AESkC: acetic acid-extracted collagen; LESkC: lactic acid-extracted collagen; CESkC: citric acid-extracted collagen.

DSC thermograms of all extracted collagens derived from the lizardfish skin (by-product) are presented in Figure 5. The results show the *T_max_* values of 40.24 °C, 38.72 °C, and 36.78 °C of AESkC, LESkC, and CESkC, respectively. The highest thermal stability was observed in the AESkC sample with a denaturation enthalpy (Δ*H*) at 1.90 J/g. For the Δ*H* point, however, the highest value was recorded in the LESkC (3.15 J/g). 

### 2.8. Microstructural Evaluation

**Figure 6:**
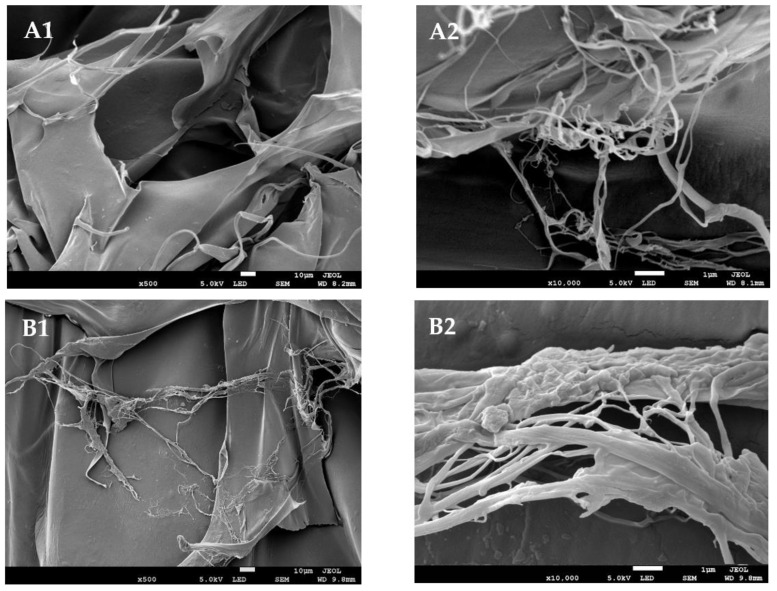

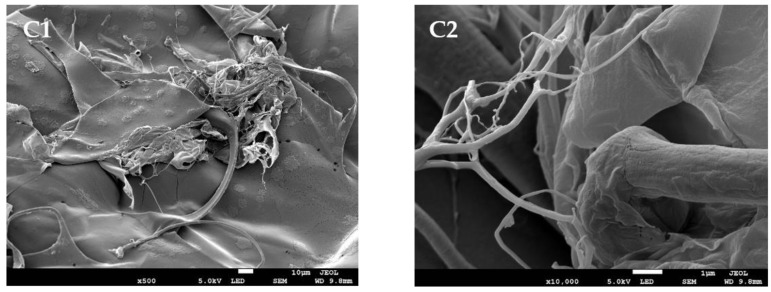
Field emission scanning electron microscopy images (FESEM) of lizardfish skin collagens extracted with different acids. (**A1**) acetic acid-extracted collagen at 500× magnification; (**A2**) acetic acid-extracted collagen at 10,000× magnification; (**B1**) lactic acid-extracted collagen at 500× magnification; (**B2**) lactic acid-extracted collagen at 10,000× magnification; (**C1**) citric acid-extracted collagen at 500× magnification; (**C2**) citric acid-extracted collagen at 10,000× magnification.

The microstructural morphology of the lizardfish collagens extracted was analyzed through FESEM images and depicted in Figure 6. Each lyophilized collagen showed a multilayer shape with irregular dense sheet-like film linked by random-coiled filaments. The fibrillar and tubular structures were obvious in the AESkC, LESkC, and CESkC samples, however, their thicknesses varied. The AESkC exhibited less thicknesses than the LESkC and CESkC. 

### 2.9. Solubility Test

**Figure 7:**
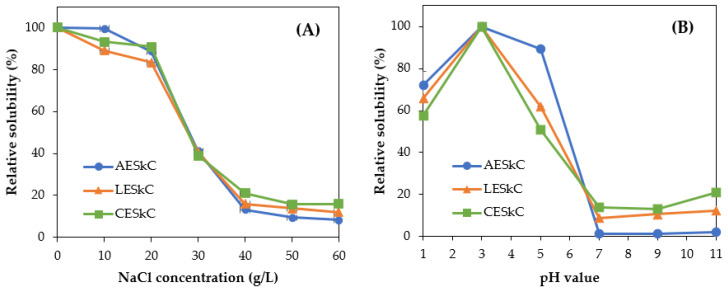
Solubility evaluation of lizardfish skin collagens extracted with different acids. (**A**) at different pH level and (**B**) at NaCl treatment. AESkC: acetic acid-extracted collagen; LESkC: lactic acid-extracted collagen; CESkC: citric acid-extracted collagen.

Figure 7A shows the effect of different pH treatments on the extracted collagens. In general, all extracted collagens were soluble in acid solutions with pHs ranging from 1.0 to 5.0. The highest solubility (*p* < 0.05) was noted at pH 3.0. However, under neutral and alkaline treatment, extracted collagens showed low solubility (<20%). In the case of the NaCl treatment (Figure 7B), similar solubility patterns were observed in the AESkC, LESkC, and CESkC. The higher solubility was recorded at low NaCl concentrations ranging from 0 g/L to 20 g/L for all collagens. In contrast, the solubility was sharply decreased (*p* < 0.05) with the addition of the NaCl concentration (more than 30 g/L).

## 3. Discussion

Collagen from fish by-products have attracted huge attention in the last few decades due to their biocompatibility, biodegradability, easy extractability, low production cost, low immunogenicity, and their safety [10]. In the present study, we extracted collagens from the skin of lizardfish using three different acids (viz., acetic acid, lactic acid, and citric acid). The microstructural and physicochemical properties of the extracted collagens were studied as well. The acid-extraction process in this experiment produced about 11.39 ± 1.05% to 11.73 ± 1.14% of collagen in comparison to sturgeon fish (*Huso huso*) (9.98%) [36], sailfish (*Istiophorus platypterus*) (5.76%) [37], and silver catfish (*Pangasius* sp.) (5.47–10.94%) [17] fish skin collagen. The lizardfish skin collagens were higher than most of the fish examined, except the bigeye tuna (*T. obesus*) (13.5%) [19] and Spanish mackerel (*Scomberomorous niphonius*) (13.68%) [38]. The variation in yields of collagens reported might be due to the types and sources of the fish species used. Besides that, extraction solvents (acids) and conditions during extraction could also influence the collagen yield [19]. In terms of hydroxyproline (Hyp) content, the AESkC showed the highest Hyp concentration (106.07 ± 0.13 mg/mg), followed by LESkC (82.42 ± 0.21 mg/g), and CESkC (80.34 ± 0.46 mg/g). These results were in agreement with Hyp contents from the marine eel-fish (*E. macrura*) (94–98 mg/g) [15], cobia (*Rachycentron canadum*) (84–99 mg/g) [39], and bigeye tuna (*T. obesus*) (82–87 mg/g) [19]. The total collagen is calculated by multiplying Hyp content with a conversion factor of 7.7 (Table 1). The various levels of Hyp (mg/g) and collagen (mg/g) as noted in the present study could be affected by several factors, such as species, size, age, and the structure and composition of fish tissue, as well as the extraction methods [40].

Color is an important parameter of collagen, since it would be applied in the development of food, nutraceutical, cosmetic, pharmaceutical, and medical products. As presented in Table 1, the significantly higher L* (brightness) value (*p* < 0.05) was observed in the CESkC sample rather than the AESkC and LESkC samples. Furthermore, the L* value of lizardfish skin collagens in the present study was greater compared to the collagen from barramundi (*Lates calcarifer*) skin (L*= 44.76–65.41) [41]. According to Gaurav et al. [42], brighter collagen is preferable in the development of new food products because there will be less or no interference with the product’s original color. In addition, all extracted collagens had a high whiteness index (WI) (72.12 ± 0.74–77.93 ± 2.53), with the highest found in the CESkC. It may be suggested that the lizardfish skin collagens have a desirable color attribute, especially the CESkC. Under UV-vis spectra (Figure 2), it has been confirmed that the extracted collagens were in line with other collagens obtained from the skin of barramundi (*L. calcarifer*) (230.3 nm) and tilapia (*O. niloticus*) (230.9 nm) [43], black ruff (*C. niger*) (232 nm) [14], and channel catfish (*Ictalurus punctaus*) (232 nm) [44]. Moreover, based on the prominent peak positions that are located at 230.0–231.5 nm, it could be assumed that the lizardfish skin collagens had a low concentration of aromatic amino acids (i.e., phenylalanine, histidine, tyrosine, and tryptophan) because these amino acids typically absorb UV lights at 250 nm and 288 nm [44]. 

Lizardfish skin collagens extracted from different acids had similar absorption peaks consisted of amide A, amide B, amide I, amide II, and amide III, as illustrated in Figure 2, and their peaks were clearly assigned in Table 2. FTIR analysis could be used for the identification of the triple-helical structures of fish collagen by measuring the absorption in the amide I-III regions. The difference in wavenumber (cm^−1^) between amides I and II can be determined using Δ*v*(v*_I_*-v*_II_*), where values < 100 cm^−1^ indicate that the triple-helical structure of collagen has been maintained [45]. The Δv values of AESkC, LESkC, and CESkC were below 100 cm^−1^, specifically 87.59 cm^−1^, 87.59 cm^−1^, and 87.60 cm^−1^, respectively. Through this determination, it has been presumed that the triple-helical structures of collagen extracted from the skin of lizardfish were preserved. Another way to verify the structure of the collagen triple helix is by using the absorption ratio (>1.0) of the amide III to the 1450 cm^−1^ band (AIII/A1450) [46]. After verification, the results also indicated that the triple-helical structures of the acid-extracted collagens were maintained because their absorption ratio values (AESkC = 1.18, LESkC = 1.17, and CESkC = 1.17) were higher than 1.0. The absorption peaks found in the lizardfish skin collagens were in accordance with previous investigations on collagen from the skin of bigeye tuna (*T. obesus*) [19], loach fish (*Misgurnus anguillicaudatus*) [47], tilapia (*O. niloticus*) [23], and sharpnose stingray (*Dasyatis zugei*) [48].

The results of the SDS-PAGE under the reducing and non-reducing conditions of the lizardfish skin collagens are presented in Figure 4. The AESkC, LESkC, and CESkC samples were categorized as type I collagen due to the presence of α_1_ and α_2_ chains. The molecular weights (MW) of α_1_ and α_2_ of AESkC, LESkC, and CESkC were generally at 139.61 kDa and 123.96 kDa, respectively. Additionally, both alpha chains had different band intensities, with a ratio of approximately 2:1. These findings were in accordance with type I collagens from the skin of sturgeon (*H. huso*) [36], loach (*M. anguillicaudatus*) [47], red stingray (*Dasyatis akajei*) [49], rohu (*Labeo rohita*) [50], and southern rays bream (*Brama australis*) [51]. The β- and γ-chains represent dimer and trimer, respectively, and were also detected in the lizardfish skin collagens (MW = 259.24 kDa and 346.56 kDa, respectively). These chains were also exhibited in grass carp (*Ctenopharyngodon idellus*) [52], bigeye tuna (*T. obesus*) [19], and spotted golden goatfish (*Parupeneus heptacanthus*) [53]. Similar electrophoretic patterns were detected in the AESkC, LESkC, and CESkC under reducing and non-reducing conditions. It could be assumed that all collagen samples used in this study did not contain disulfide bone (R−S−S−R′). Furthermore, the X-ray diffraction (XRD) feature of the collagens from the lizardfish skin was similar to the previous reports on three genetic lines of tilapia (*O. niloticus*) [54], Nile tilapia (*O. niloticus*) [55], and carp fish (*Ctenopharyngodon idellaarpio*) [56]. All extracted collagens (AESkC, LESkC, and CESkC) possessed two peaks at diffraction angles (2θ) of 7.44° and 20.86°, 7.40° and 20°, and 7.63° and 19.17°, respectively. The first sharp peak (peak A) is closely related to the triple-helical structure of collagen. To measure the minimum value of the repeated spacings (d (Å)), the Bragg equation d(Å) = λ/2sinθ (where λ is the X-ray wavelength (1.54 Å) and θ is the Bragg diffraction angle) was applied [50]. The d values of the first relatively sharp peak, indicating the distance between the molecular chains, were between 11.87 Å and 11.92 Å, and those of the second broad peak, reflecting the distance between skeletons, were ranging from 4.25 Å to 4.63 Å. These results correspond to the diameter of a collagen molecule with a triple-helical structure and a single left-handed helix chain. Therefore, both extracted collagen samples were in their native conformations and undenatured.

The range of the *T_max_* values of the lizardfish skin collagens (from 36.78 °C to 40.24 °C) were confirmed by the DSC thermograms. A higher thermal stability was observed in the AESkC (Figure 6) and this might be due to the high content of Hyp. As presented in Table 1, the Hyp content of AESkC (106.07 mg/g) was significantly higher (*p* < 0.05) than LESkC (82.42 mg/g) and CESkC (80.34 mg/g). According to Benjakul et al. [57], the thermal stability of triple-helical collagen was structured by the pyrrolidine rings of imino acid (hydroxyproline and proline), and it was partially formed by the hydrogen (H) bonding through the hydroxyl group of hydroxyproline. Additionally, hydroxyproline might stabilize the triple-helical structure of collagen via hydrogen bonding in coil-coiled α chains [58]. In comparison to previous reports of the fish skin collagens, such as rohu (*L. rohita*) (*T_max_* = 36.40 °C) [50], loach (*M. anguillicaudatus*) (*T_max_* = 36.03 °C) [47], grass carp (*C. idellus*) (*T_max_* = 35.60 °C) [52], and bigeye snapper (*Priancanthus tayenus*) (*T_max_* = 31.48 °C) [59], the *T_max_* values of the lizardfish skin collagens were slightly greater, particularly in the AESkC. It could be proposed that the helical secondary structures of collagen extracted from the skin of lizardfish was still maintained under high thermal conditions. For the Δ*H* value, the widest area under the peaks was exhibited in the LESkC (3.15 J/g), indicating the highest energy required to uncouple the α-chains of lactic acid-extracted collagen and convert them into random coils, compared to that of acetic acid and citric acid-extracted fish skin collagens. However, the difference in the thermal stability of fish collagen depends on the imino acid composition, extraction step, and other environmental factors (habitat and temperature) [59]. For the microstructural study, the AESkC, LESkC, and CESkC showed irregular dense sheet-like film linked by random-coiled filaments. The loose, porous, and wrinkled structures were also found in the lyophilized samples due to the dehydration process during freeze-drying. In addition, fibrillar and tubular structures were also exhibited (Figure 7). This morphological structure of the lizardfish skin collagens was similar to collagens from the skin of black ruff (*C. niger*) [14], the skin of silver catfish (*Pangsius* sp.) [17], the scales of the miiuy croaker (*M. miiuy*) [16], and the skin of marine eel-fish (*E. macrura*) [12]. Understanding the microstructure of collagen material is an essential point for the development of collagen-based products. Therefore, many researchers recommended that collagens with interconnectivity, fibrillary, and sheet-like film structures have the potential to be used in new tissue formation, cell seeding, growth, wound healing, mass transport, and migration [16,35].

**Figure 8:**
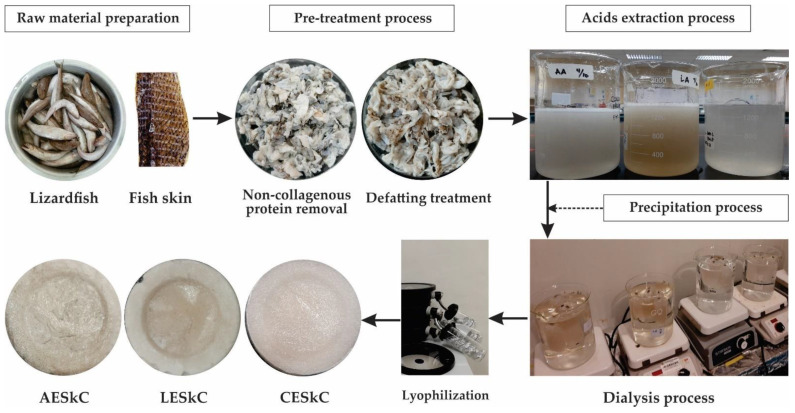
Extraction process of collagens from the skin of lizardfish. AESkC: acetic acid-extracted collagen; LESkC: lactic acid-extracted collagen; CESkC: citric acid-extracted collagen.

Similar solubility patterns were demonstrated by all extracted collagens at different pH and NaCl concentrations (Figure 8). In terms of pH, the high solubility of AESkC, LESkC, and CESkC was generally exhibited at acidic conditions (pH 1.0–5.0), with the highest solubility recorded at pH 3.0. At neutral and alkaline conditions, however, the solubility of collagen sharply decreased (<20%). This might be due to an increase in hydrophobic–hydrophobic interactions among collagen molecules, particularly at the isoelectric point (pI) [25]. The solubility profile of lizardfish skin collagens at different pH levels was similar with that of collagens from tilapia (*O. niloticus*) skin [23], horse mackerel (*Trachurus japonicus*) scale [60], and Spanish mackerel (*S. niphonius*) skin [38]. Furthermore, for NaCl treatments, the higher solubilities (>80%) of AESkC, LESkC, and CESkC were observed at low concentrations of sodium chloride (up to 20 g/L). The solubility of all extracted collagens prominently declined at higher concentrations of NaCl (>30 g/L). These results were in accordance with collagen from spotted golden goatfish (*P. heptacanthus*) [53], eel fish (*E. macrura*) [15], and golden pompano (*Trachinotus blochii*) [61]. The low solubility during the NaCl treatment might be due to the salting-out process. The higher salt concentration would increase hydrophobic interactions within polypeptide chains. In addition, competition with salt ions for water will also increase and subsequently lead to protein precipitation [23].

## 4. Materials and Methods

### 4.1. Materials

Lizardfish (*Saurida tumbil*) were purchased from a wet market in Kota Kinabalu, Sabah, Malaysia. Skins of lizardfish were mechanically removed by a deboner machine (SFD-8, Taiwan) and washed with running tap water. The skin samples were cut to about 1.0 × 1.0 cm^2^ with a stainless-steel scissor (Brisscoes, Malaysia) and stored in polyethylene containers at −20 °C. Sodium dodecyl sulphate (SDS), *N,N,N′,N′*-tetramethyl ethylene diamine (TEMED), Coomassie Blue R-250, Lowry reagent, Folin–Ciocalteu’s phenol reagent, and acetic acid were supplied from Merck (Darmstadt, Germany). Molecular weight markers (dual color standards) and acrylamide purchased from Bio-Rad Laboratories (Hercules, CA, USA). Bovine serum albumin (BSA) and tris(hydroxymethyl) aminomethane hydrochloride were delivered from Sigma Chemical Co., (St. Louis, MO, USA). Other chemicals and reagents used in this study were of analytical grade. 

### 4.2. Preparation of Acid-Extracted Collagen from Lizardfish Skin

The extraction process of lizardfish skin collagen was conducted according to the method established by Matmaroh et al. [53] with slight modification. All procedures were performed in a cold room (4 °C) and each extraction step is depicted in Figure 8. Prepared skin samples were suspended in 0.1 M NaOH at the ratio of 1:10 (*w*/*v*) for 6 h with continuous stirring to remove non-collagenous proteins and pigment, and the alkaline solution was changed every 3 h. Treated samples were washed with cold distilled water to reach the neutral pH (7.0). Next, the skin samples were defatted with 10% butyl alcohol at the ratio of 1:10 (*w*/*v*) for 24 h, and the solution was changed every 12 h. The treated samples were rinsed with chilled distilled water for 30 min, and the water was changed every 10 min. Then, defatted lizardfish skins were subjected to acids-aided extraction with 0.5 M acetic, lactic, and citric acid for 72 h. After extraction, each mixture was filtered through a double layer of cheese cloth. The supernatant was precipitated by adding sodium chloride to obtain the final concentration of 2.5 M containing 0.05 M Tris (hydroxymethyl) aminomethane (pH 7). The precipitated samples were then centrifuged at 15,000× *g* for 30 min and the pellets were dissolved with 0.5 M acids at a ratio of 1:5 (*w*/*v*). The solubilized samples were dialyzed using dialysis tubing cellulose membrane (flat width 43 mm, Sigma) in 20 volumes of 0.1 M acids (acetic, lactic and citric), followed by chilled distilled water for 72 h. After dialysis, the samples were dried using a freeze-dryer (Labconco, Kansas City, MO, USA). The lyophilized acid-extracted collagens were then stored in a freezer (−20 °C) until further analyses. 

### 4.3. Yield and Hydoxyproline Determination

Yield of acid-extracted collagens from the lizardfish skin was determined based on the wet weight of raw material used in this study:

(1) $$\;\mathrm{Yield}\;\;(\%)=\frac{\mathrm{Weight}\;\mathrm{of}\;\mathrm{lyophilized}\;\mathrm{collagen}}{\mathrm{Weight}\;\mathrm{of}\;\mathrm{initial}\;\mathrm{wet}\;\mathrm{lizardfish}\;\mathrm{skin}}\times \;100$$

Hydroxyproline (Hyp) content was determined according to the method developed by Bergman and Loxley [62]. Lyophilized collagens were hydrolyzed with 6 M HCl at 110 °C for 24 h. The hydrolyzed samples were filtered through Whatman No. 4 filter paper. The filtrate was then neutralized with 5 M and 2.5 M NaOH to achieve the pH 6.0–6.5. Next, about 0.2 mL of the neutralized samples was pipetted into each glass test tube and 0.4 mL isopropanol was added. The mixtures were then added with 0.2 mL of oxidant solution and allowed to stand for 5 min at room temperature. After that, 2.3 mL of Ehrlich’s reagent solution was added and mixed well. Subsequently, the tubes were heated at 60 °C for 25 min in a water bath (Memmert, Schwabach, Germany). The heated solutions were then cooled for 5 min in chilled water and diluted to 10 mL with isopropanol. Absorbance against water was measured at 558 nm. The Hyp standard solution (10 to 70 ppm) was also determined.

### 4.4. Colour Analysis

Colour analysis of extracted collagens was performed according to the method described by Huda et al. [7] using with a colorimeter (ColorFlex CX2379, HunterLab, Galveston, TX, USA). Attributes of color tested include lightness (L*), redness (a*), and yellowness (b*). Whiteness index (WI) was calculated based on the study of Briones and Anguilera [63] using the following equation:

(2) $${\mathrm{WI}=100\;-\;[(100\;-\;L*)}^{2}+\left({a*}^{2}\right){+(b*}^{2}{)]}^{0.5}$$

### 4.5. Sodium Dodecyl Sulfate-Polyacrylamide Gel Electrophoresis (SDS-PAGE)

SDS-PAGE was carried out using the method of Laemmli [64] with some modifications, using a Mini-PROTEAN electrophoresis system (Bio-Rad Laboratories, Hercules, CA, USA). The lyophilized collagens (2.5 mg/mL) were dissolved in SDS solution (5%) and mixed well. The mixtures were then heated at 85 °C for 1 h in a water bath (Memmert, Schwabach, Germany). After heat treatment, the samples were centrifugated at 8500× *g* for 5 min at room temperature to remove undissolved debris. The solubilized samples were mixed with the same volume of sample buffer (0.5 M Tris–HCl, pH 6.8, containing 4% SDS and 20% glycerol) in the presence and absence of 10% β-mercaptoethanol, and then heated for 3 min. Next, approximately 15 µL of each collagen sample (10 µg protein) was loaded onto a polyacrylamide gel consisting of a 7.5% resolving gel and 4% stacking gel. Electrophoresis process was set at a constant voltage of 120 V for 1.5 h, and the gel was fixed with 50% (*v*/*v*) methanol and 10% acetic acid for 10 min. Then, the fixed gel was stained for 10 min with 0.05% (*w*/*v*) Coomassie blue R-250 in 5% (*v*/*v*) acetic acid and 15% (*v*/*v*) methanol. After staining, the gel was destained with 30% (*v*/*v*) methanol and 10% (*v*/*v*) acetic acid. The molecular weight markers were determined using a prestained natural protein standards (dual color standards) (Bio-Rad Laboratories, Hercules, CA, USA). 

### 4.6. Ultraviolet-Visible Spectroscopy (UV-Vis)

UV absorption spectrum of lizardfish skin collagens was derived from a UV-Vis spectrophotometer (Agilent Cary 60, Cary, NC, USA). Each extracted collagen (10 mg) was dissolved in 1 mL of 0.5 M acetic acid, and the sample solution was dropped into a quartz cell with a path length of 1 mm. Spectrum was determined at wavelengths between 400 nm and 200 nm and the baseline used in this analysis was 0.5 M acetic acid solution [39]. 

### 4.7. Attenuated Total Reflectance–Fourier Transform Infrared Spectroscopy (ATR–FTIR)

ATR-FTIR spectra of extracted collagens were determined using a FTIR spectrometer apparatus (Agilent Cary 630, Cary, NC, USA). The procedure was adopted from a previous study by Matmaroh et al. [53]. Around 5 mg of lyophilized samples were placed into the crystal cell of spectrometer. All spectra were run within the wavenumber range of 4000–400 cm^−1^ with a resolution of 2 cm^−1^ for 32 scans against a background spectrum recorded from the clean empty cells at room temperature. Spectra data were measured using the Agilent Microlab software program.

### 4.8. X-ray Diffraction (XRD)

XRD of each extracted skin collagen was carried out according to the method described by Chen et al. [23]. The prepared samples were scanned using an XRD instrument (Rigaku Smart Lab^®^, Tokyo, Japan) with copper Kα as a source of X-rays. The tube voltage and current were set at 40 kV and 40 mA, respectively. The scanning range was determined to be between 10° and 50° (2θ) with a speed of 0.06° per second.

### 4.9. Differential Scanning Calorimetry (DSC)

DSC of acid-extracted skin collagens was conducted following the procedure described by Kittiphattanabawon et al. [59]. The freeze-dried samples were rehydrated with deionized water at a solid/solution ratio of 1:40. The rehydrated samples were then allowed to stand for 2 days in a chiller (4 °C) and weighed accurately into aluminum pans (6–12 mg) and sealed tightly. Before scanning, DSC instrument (Perkin-Elmer, Model DSC7, Norwalk, CA, USA) was calibrated using indium as a standard. Then, the sealed samples were scanned between the range of 20 °C and 50 °C with heating rate at 1 °C/min. An empty pan was prepared for the reference. The maximum transition temperature (*T_max_*) was detected from the endothermic peak of thermogram. Total denaturation enthalpy (Δ*H*) was recorded by measuring the area of the DSC thermogram.

### 4.10. Field Emission Scanning Electron Microscopy (FESEM)

Microstructural characteristics of lizardfish skin collagens extracted with acetic, lactic, and citric acids were studied by FESEM using S-4800 FESEM machine (Hitachi, Japan). Lyophilized samples were sputter coated for 5 min with gold using a JEOL JFC-1200 (Tokyo Rikakikai Co., Ltd., Tokyo, Japan) fine coater. 

### 4.11. Solubility of Lizardfish Skin Collagens

Solubility test of all extracted collagens was treated with different pH values and the NaCl concentrations were measured using the previous method [60]. For pH evaluation, the prepared collagens were suspended overnight in 0.5 M acetic acid solution with continuous stirring at 4 °C. Next, the mixtures were subjected to adjustment at different pH levels between 1.0 and 11.0 using 2.5 N NaOH and 2.5 N HCl solutions. The pH-adjusted samples were then allowed to stand for 1 h and centrifuged at 8500× *g* for 30 min in the Eppendorf 5430R Refrigerated Centrifuge (Hampton, VA, USA). In terms of NaCl treatment, the concentrations used in this study were in the range of 0–60 g/L. Five milliliters of solubilized samples were mixed with 5 mL of NaCl solution. The mixtures were then stirred at 4 °C for 1 h using a FAVORIT Magnetic Stirrer ST0707V2 (Selangor, Malaysia). Then, the mixtures were centrifuged at 8500× *g* for 30 min at 4 °C. Protein content in the solubilized samples was determined using the established method [65] with bovine serum albumin (BSA) as a standard. Relative solubility values of both treatments were calculated using the following formula:

(3) $$\mathrm{Relative}\;\mathrm{solubility}\left(\%\right)=\frac{\mathrm{Current}\;\mathrm{concentration}\;\mathrm{of}\;\mathrm{protein}\;\mathrm{at}\;\mathrm{current}\;\mathrm{pH}}{\mathrm{The}\;\mathrm{highest}\;\mathrm{concentration}\;\mathrm{of}\;\mathrm{protein}}\;\times \;100$$

(4) $$\mathrm{Relative}\;\mathrm{solubility}\left(\%\right)=\frac{\mathrm{Current}\;\mathrm{concentration}\;\mathrm{of}\;\mathrm{protein}\;\mathrm{at}\;\mathrm{current}\;\mathrm{NaCl}}{\mathrm{The}\;\mathrm{highest}\;\mathrm{concentration}\;\mathrm{of}\;\mathrm{protein}}\;\times \;100$$

### 4.12. Statistical Analysis

Experiments were performed in triplicate and the data were expressed as the means ± standard deviation. One-way ANOVA was performed, and mean comparisons were analyzed by Duncan’s multiple range tests using SPSS Statistics version 28.0 (IBM Corp., Armonk, NY, USA).

## 5. Conclusions

Collagen type I could be extracted from the skin of lizardfish (*S. tumbil*) using acetic, lactic, and citric acids. Acetic acid-extracted collagen (AESkC) exhibited a higher yield, compared to LESkC and CESkC, although it was not significantly different (*p* > 0.05). Besides, AESkC also had the highest hydroxyproline content, which possibly contributed to the relatively high thermal stability. Moreover, the triple-helical structure of all extracted collagens was maintained as observed under the FTIR spectra and the X-ray diffraction test. We may conclude that the type I collagen extracted from lizardfish skin could be posed as an alternative collagen source. 

## Figures and Tables

**Table 1:** Yield, Hyp, collagen, and color analysis of the lizardfish skin collagen extracted with various organic acids.

Sample	Yield (%)	Hyp (mg/g)	Collagen (mg/g)	Colour Attributes			
				L*	a*	b*	WI
AESkC	11.73 ± 1.14 ^a^	106.07 ± 0.13 ^c^	816.77 ± 1.00 ^c^	72.76 ± 1.20 ^a^	1.32 ± 0.14 ^a^	4.91 ± 0.13 ^a^	72.29 ± 1.21 ^a^
LESkC	11.63 ± 1.10 ^a^	82.42 ± 0.21 ^b^	634.62 ± 1.65 ^b^	73.17 ± 0.67 ^a^	2.09 ± 0.30 ^b^	7.30 ± 0.27 ^b^	72.12 ± 0.74 ^a^
CESkC	11.39 ± 1.05 ^a^	80.34 ± 0.46 ^a^	618.61 ± 3.52 ^a^	78.52 ± 2.74 ^b^	1.33 ± 0.16 ^a^	4.75 ± 0.70 ^a^	77.93 ± 2.53 ^b^

Values are given as mean ± standard deviation from triplicate determinations (*n* = 3). Means in the same column with different superscripts (a, b and c) are significantly different (*p* < 0.05). AESkC: acetic acid-extracted collagen; LESkC: lactic acid-extracted collagen; CESkC: citric acid-extracted collagen; Hyp: hydroxyproline content. * indicates that this is the new colour system; it is the follow-up of the older CIELAB system.

**Table 2:** Fourier transform infrared spectroscopy peak locations and the assignment for collagens from the skin of lizardfish.

Peak Location			Peak Assignment	References
AESkC	LESkC	CESkC		
3278.28	3296.91	3285.73	Amide A: mainly N-H stretching coupled with hydrogen bond	[33]
3071.4	3073.27	3086.31	Amide B: CH_2_ asymmetric stretching	[33]
1628.89	1628.89	1628.89	Amide I: C=O stretching/hydrogen bond coupled with COO-	[34]
1541.29	1541.29	1541.29	Amide II: N-H bend coupled with C-N stretching	[35]
1233.78	1235.64	1237.51	Amide III: N-H bend coupled with C-H stretching	[34]

AESkC: acetic acid-extracted collagen; LESkC: lactic acid-extracted collagen; CESkC: citric acid-extracted collagen.

## Data Availability

The data presented in this study are available upon request from the corresponding author.
